# A Challenge-Oriented Review of Delivery Systems for Cell and Gene Therapies in Intervertebral Disc Degeneration

**DOI:** 10.3390/bioengineering13050566

**Published:** 2026-05-16

**Authors:** Wenbo Wu, Zhangrong Cheng, Haiyang Gao, Xianglong Chen, Wang Wu, Zimu Yu, Cao Yang, Yukun Zhang

**Affiliations:** Department of Orthopedics, Union Hospital, Tongji Medical College, Huazhong University of Science and Technology, Wuhan 430022, China

**Keywords:** intervertebral disc degeneration, bioengineered delivery systems, cell and gene therapy, challenge-oriented design, translational barriers

## Abstract

Intervertebral disc degeneration (IVDD) is the leading cause of low back pain, a global public health burden for which current pharmacological and surgical treatments provide symptomatic relief but fail to reverse the underlying degenerative process. The uniquely avascular, hypoxic, acidic, and mechanically demanding disc microenvironment poses formidable barriers to the survival and function of therapeutic cells and genes, emphasizing the critical need for bioengineered delivery systems. In this review, we introduce the structure and microenvironment of the intervertebral disc, as well as the molecular mechanisms underlying IVDD. We then provide a critical comparative analysis of delivery platforms, including hydrogels, microspheres, nanoparticles, nanofibrous scaffolds, and viral and non-viral vectors, around five core delivery challenges: mechanical protection, retention and leakage prevention, targeted intracellular delivery, controlled release kinetics, and metabolic support. Furthermore, we examine the fabrication technologies and material considerations that determine platform performance, and we analyze the translational barriers that have impeded clinical adoption, such as the limitations of small-animal models and unresolved cell leakage. Finally, we highlight emerging strategies, including gene-cell combination therapy and endplate preconditioning, to accelerate the clinical translation of precision therapies for IVDD.

## 1. Introduction

Low back pain (LBP) is a prevalent musculoskeletal disorder worldwide, representing a leading cause of years lived with disability and imposing a substantial socioeconomic burden [1,2]. Intervertebral disc degeneration (IVDD) has been identified as the most common etiology of LBP, characterized by a progressive imbalance between extracellular matrix (ECM) anabolism and catabolism, loss of resident nucleus pulposus cells (NPCs), and chronic inflammatory cascades [3,4]. Current clinical management, including pharmacological interventions and surgical procedures, provides symptomatic relief but fails to reverse the underlying degenerative process. Furthermore, they cannot restore the disc’s native structure or mechanical function [5,6]. To overcome these obstacles, it is urgent to pursue effective therapeutic strategies to regenerate and restore disc function.

Advances in understanding IVDD pathophysiology have spurred the development of cell-based and gene-based therapies aimed at directly replenishing depleted cell populations or intervening in resident cells to reestablish ECM homeostasis [7,8]. A deeper understanding of the pathophysiology of IVDD has driven the development of cell- and gene-based therapies aimed at replenishing depleted cell populations and restoring extracellular matrix (ECM) homeostasis. Pathological mechanisms, including elevated matrix-degrading enzymes, increased inflammatory cytokine levels, and accelerated apoptosis, have been identified as a series of therapeutic targets for intervention [9,10,11]. First, catabolic enzymes can be directly inhibited by delivering TIMP genes or using Genetic depletion of ADAMTS-4/5, while anti-inflammatory miRNAs such as miR-128-3p can block cytokine-driven feedback loops [12,13,14]. Second, the IL-1Ra gene can be delivered, or NF-κB can be targeted to block intracellular inflammatory signaling and counteract pro-inflammatory cytokines at the receptor level [15,16]. Concurrently, targeting growth factors or corresponding transcription factor genes can promote anabolic processes to stimulate ECM synthesis [17,18,19,20,21]. At the same time, cell therapy addresses the loss of resident cells by replenishing various types of stem cells and NPCs; these cells not only provide a cellular source for matrix regeneration but also exert paracrine anti-inflammatory and immunomodulatory effects [22,23,24,25].

However, translating these therapeutic strategies into clinical reality requires overcoming a series of formidable, interconnected delivery bottlenecks imposed by the uniquely harsh disc microenvironment [26]. The intervertebral disc (IVD) is characterized by avascularity, severe hypoxia, acidic pH, high osmolarity, and complex multiaxial mechanical loading, with intradiscal pressures reaching 0.5–3.0 MPa during lifting [4,27,28,29]. These conditions present multiple core delivery challenges, which form the central focus of this review: (1) Mechanical protection: The delivery system must protect the encapsulated cargo from high mechanical forces while matching the modulus of the native tissue to provide support for restoring disc height. (2) Retention and leak prevention: Post-injection leakage is a major cause of complications such as ectopic osteophyte formation. Therefore, the delivery system must achieve rapid in situ solidification and tissue adhesion to ensure effective retention [30]. (3) Targeted intracellular delivery: Gene therapy vectors must overcome various biological barriers to achieve efficient cellular uptake and sustained transgene expression. (4) Controlled release kinetics: Maintaining therapeutic efficacy in chronic, slowly progressive diseases requires temporal control of payload release to match the IVDD’s recovery cycle, which ranges from weeks to months. (5) Metabolic support: Degenerated intervertebral discs are nutritionally deprived. If metabolically active cells are implanted into this environment, necrosis may be accelerated unless the delivery system provides a diffusible porous structure, prioritizes paracrine nutrition, or incorporates oxygen-producing materials.

In this review, we move beyond traditional material-based classifications to provide a critical comparative analysis of delivery platforms organized around these five core challenges. We introduce the structure and microenvironment of the intervertebral disc, the molecular mechanisms driving IVDD, and the current diagnostic and therapeutic landscape. Furthermore, we examine the fabrication technologies and material considerations that determine platform performance. Finally, we summarize the latest strategies in cell-based and gene-based delivery for IVDD, evaluate translational barriers that impede clinical application, and highlight emerging strategies for clinical translation. This platform-centered, challenge-oriented review aims to guide the rational design of next-generation precision delivery systems for IVDD.

## 2. Intervertebral Disc Structure and Degeneration Mechanisms

### 2.1. Intervertebral Disc Structure and Microenvironment

**Figure 1:**
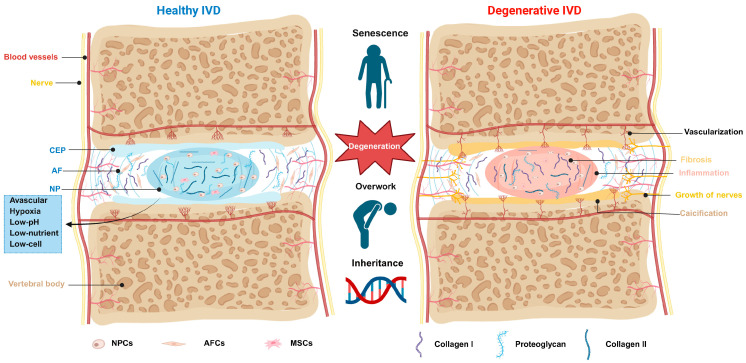
Schematic highlighting the structure and microenvironment of the normal intervertebral disc compared to the degenerative IVD. The main pathological and morphological changes are inflammatory response, ingression of blood vessels and nerves, fibrosis of IVD tissue, and endplate calcification.

The IVD is a tripartite structure comprising the central nucleus pulposus (NP), the surrounding annulus fibrosus (AF), and the cartilaginous and bony endplates (CEPs and BEPs) that interface with the adjacent vertebral bodies (Figure 1) [6]. Each compartment harbors a distinct cell population that synthesizes a compartment-specific ECM, collectively establishing the unique microenvironment essential for disc mechanical function [27].

Nucleus pulposus cells (NPCs) produce a highly hydrated ECM rich in proteoglycans and type II collagen, endowing the NP with the capacity to absorb and dissipate axial compressive loads [31]. The AF is organized into two concentric regions: an outer zone of dense, highly oriented type I collagen lamellae and a less ordered inner zone. Annulus fibrosus cells (AFCs), which exhibit a fibroblast-like morphology, provide the tensile strength necessary to contain NP osmotic pressure and resist bending, torsion, and shear [32,33]. The endplates serve dual mechanical and nutritional roles. They distribute intradiscal pressure to the vertebral bodies and serve as the primary conduit for nutrient diffusion from vertebral capillaries to the avascular disc [34,35].

Because the IVD lacks an intrinsic capillary network, cellular metabolism depends entirely on the diffusion from capillaries in the endplates and the outermost AF. This arrangement creates steep concentration gradients within the disc: glucose concentrations in the central NP fall below 0.5 mM, and the partial pressure of oxygen decreases to approximately 1% [6]. The resulting reliance on anaerobic metabolism drives lactate accumulation. It acidifies the extracellular milieu [36,37], while age-related endplate calcification progressively restricts solute transport, further compromising the nutritional supply and waste removal [38]. The disc is therefore characterized by a uniquely harsh microenvironment defined by avascularity, severe hypoxia, low pH, high osmolarity, and complex multiaxial mechanical loading.

Despite these challenges, resident NPCs exhibit adaptive mechanisms. Hypoxia-inducible factor (HIF) plays a central regulatory role in NPC survival, matrix synthesis, and apoptosis. A defining feature of NPCs is their capacity to stabilize HIF-1α function even under non-hypoxic conditions [39,40]. This microenvironment can also induce cell differentiation. In a three-dimensional in vitro model, rat mesenchymal stem cells (MSCs) exposed to TGF-β1 under hypoxic conditions (2% O_2_) acquired an NP-like phenotype, with upregulated expression of Sox-9, aggrecan, and type II collagen, demonstrating that the niche can direct chondrogenic differentiation [41]. Nevertheless, prolonged exposure to these metabolic and mechanical stresses ultimately overwhelms adaptive mechanisms, promoting cellular senescence and apoptosis.

Additional constraints include hypocellularity and slow cell turnover. The normal NP contains approximately 5  ×  10^6^ cells/cm^3^, and the AF approximately 9  ×  10^6^ cells/cm^3^, and these numbers decline further with aging and degeneration [42,43,44]. The combination of low cell density, limited nutrient supply, and high biomechanical demand creates an environment with a low injury threshold and severely limited self-repair capacity. Therefore, a precise understanding of the microenvironment is crucial for the design of therapeutic delivery systems.

### 2.2. Mechanisms of IVDD

At the cellular and molecular level, IVDD is a process of progressive ECM metabolic imbalance [45]. During this process, catabolic activity exceeds anabolic activity, leading to matrix degradation, dehydration, and mechanical dysfunction. Multiple contributing factors, including genetic susceptibility, abnormal mechanical loading, endplate calcification, and cellular senescence, converge on a common pathological pathway: upregulation of matrix-degrading enzymes (MMP-1, MMP-3, MMP-13, ADAMTS4, ADAMTS5), elevated inflammatory cytokines (IL-1β, TNF-α, IL-6), diminished matrix synthesis, and accelerated cell death [46,47]. Aggrecan cleavage reduces NP osmotic pressure, causing height loss and impaired load distribution. Concurrently, disrupted collagen within the AF facilitates annular tears, radial fissures, and disc herniation [38].

Inflammatory mediators exacerbate this cascade, cytokines further stimulate MMP/ADAMTS expression, inhibit matrix protein synthesis, and promote neurotrophic and angiogenic factors, thereby facilitating nociceptive nerve and blood vessel ingrowth into the aneural, avascular disc [35]. This neurovascular invasion and ECM breakdown products establish a self-perpetuating degenerative cycle. Furthermore, oxidative stress, DNA damage, and endoplasmic reticulum stress drive IVD cells’ senescence and apoptosis [10,46,48,49], while autophagy is impaired, rendering cells increasingly vulnerable. These combined factors lead to the gradual loss of matrix-producing cells, ultimately making the disc incapable of self-repair.

### 2.3. Diagnosis, Staging, and Clinical Management of IVDD

**Figure 2:**
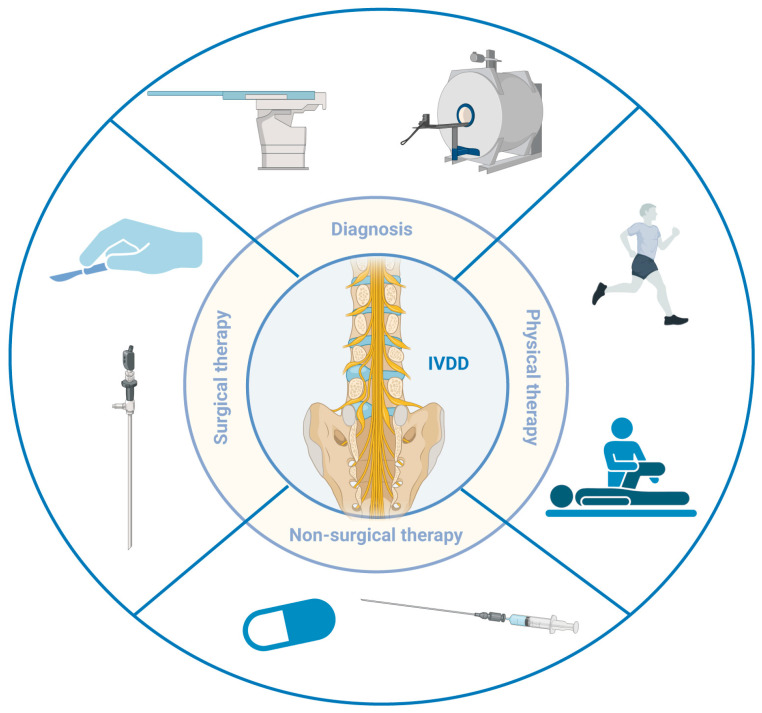
Diagnosis and current IVDD treatments (Surgical/Non-surgical therapy, Physical therapy).

Understanding the molecular and cellular mechanisms described above provides the biological rationale for intervention, but translating this knowledge into clinical therapy first requires precise assessment of disease severity and stage (Figure 2). Clinically, the diagnosis of IVDD integrates three levels of evidence: patient history, physical examination, and imaging findings. The cardinal symptom is mechanical low back pain that worsens with sitting, bending, coughing, and weight-bearing [50]. When degeneration leads to nerve root compression, radicular pain, sensory disturbance, and motor weakness may develop in the corresponding dermatomal and myotomal distributions.

Imaging remains the primary method for evaluating IVDD. X-ray provides an initial survey of disc height loss, osteophyte formation, endplate sclerosis, and segmental instability but lacks sensitivity for early degenerative changes [51]. Computed tomography (CT) offers superior visualization of bony anatomy and calcification patterns, including endplate sclerosis and posterior element pathology [52]. Magnetic resonance imaging (MRI) is the current gold standard owing to excellent soft-tissue contrast, which enables simultaneous evaluation of disc hydration, annular integrity, endplate signal changes, and neural compression [53,54].

The most widely adopted semi-quantitative grading system is the Pfirrmann classification, which assigns degenerative severity into five grades based on T2-weighted signal characteristics and disc structural features [55]. Grade I represents a normal disc with homogeneous hyperintense signal and preserved height; Grade II shows inhomogeneous hyperintensity with a horizontal hypointense band; Grade III is characterized by intermediate gray signal with an indistinct nucleus-annulus boundary; Grade IV demonstrates moderate signal loss with collapsed disc space; and Grade V indicates severe signal loss with complete disc space collapse. The inter-observer reliability of Pfirrmann grading, expressed as kappa values ranging from 0.69 to 0.81, indicates substantial agreement [56,57]. Yet, the distinction between Grades II and III remains a principal source of variability because it depends on the subjective interpretation of signal heterogeneity. Moreover, Pfirrmann grading primarily reflects water content and gross morphology and may not capture early functional deterioration that precedes visible signal change. Complementing Pfirrmann grading, Modic changes describe vertebral bone marrow signal alterations adjacent to the endplates and provide additional information on the biological activity of degeneration [58]. Modic Type I (T1-hypointense, T2-hyperintense) indicates active bone marrow edema and inflammation and is strongly associated with pain; Type II signifies fatty marrow replacement; Type III represents endplate sclerosis. The presence of inflammatory Modic Type I changes portends impaired nutrient diffusion across the endplate. This damage directly affects the efficacy of any intradiscal delivery system.

Clinical management is stratified according to symptom severity and structural degeneration [59,60]. The majority of patients do not require surgical intervention. First-line treatment aims to relieve pain and improve function through non-pharmacologic and pharmacologic modalities. Non-pharmacologic approaches include structured exercise, physical therapy, and weight management. Pharmacologic management relies primarily on nonsteroidal anti-inflammatory drugs (NSAIDs), and, in select cases, muscle relaxants, opioid analgesics, or epidural steroid injections. Early-stage patients treated with appropriate functional exercise combined with oral anti-inflammatory and analgesic medications frequently achieve substantial pain relief and slowed disease progression. In advanced cases presenting with radicular symptoms, local corticosteroid or anesthetic injections may provide both therapeutic benefit and diagnostic confirmation. However, NSAIDs carry risks of gastrointestinal and antiplatelet adverse effects, and opioids pose addiction liability [61].

When conservative measures fail or when progressive neurological deficits occur, surgical intervention becomes necessary. Options include discectomy, laminectomy with decompression, and spinal fusion [62]. Although these procedures provide rapid relief of neuralgic pain, they are associated with adjacent segment degeneration, postoperative low back pain, and loss of lumbar lordosis [63,64]. Percutaneous endoscopic discectomy offers reduced tissue trauma, shorter hospitalization, and accelerated recovery, yet it addresses only the symptomatic disc portion and carries a finite recurrence rate. Critically, none of the current pharmacologic or surgical strategies target the underlying biological drivers of degeneration. This fundamental therapeutic gap underscores the urgent need for more optimal and valuable therapeutic approaches to repair or even reverse the degenerative changes in the disc.

### 2.4. Mechanistic Mapping of Therapeutic Targets

The pathological mechanisms underlying the mentioned inflammatory cytokine cascade and ECM degradation identify a series of actionable targets for therapeutic intervention. Matching these targets with specific therapeutic strategies is crucial for the rational design of drug delivery systems. First, catabolic enzymes are direct targets for inhibition. Delivery of TIMP genes can directly counteract MMP activity, while siRNA-mediated silencing of ADAMTS-4/5 prevents glycoprotein degradation [65,66]; anti-inflammatory miRNAs, such as miR-146a, can block cytokine-driven positive feedback loops by targeting upstream signaling adaptors, such as TRAF6 [67,68,69,70]. Pro-inflammatory cytokines themselves can be antagonized at the receptor level, for example, by delivering the IL-1Ra gene or by targeting NF-κB to block its intracellular inflammatory signaling pathways [16,71]. Concurrently, delivering growth factor genes (including BMP-2, BMP-7, GDF-5, and TGF-β3) or their corresponding transcription factors (such as Sox-9 and LMP-1) to promote anabolic processes can stimulate ECM synthesis, thereby promoting the chondrogenic differentiation phenotype of intervertebral disc cells [22,72,73,74,75]. Cell-based therapies, however, target the primary characteristic of IVDD-the loss of resident cells. The supplementation of MSCs not only provides a cellular source for matrix generation but also offers paracrine nutritional support, which can stimulate endogenous cells and exert anti-inflammatory and immunomodulatory effects [76,77,78,79].

Importantly, these therapeutic strategies are not exclusive. The multifactorial pathology of IVDD suggests that combined treatment regimens may be necessary to achieve sustained functional recovery. This mechanistic map, which links each pathological node to a specific therapeutic strategy, provides a conceptual framework for guiding the choice and design of delivery systems discussed in subsequent sections.

After identifying the key pathological mechanisms underlying IVDD and linking them to specific therapeutic strategies such as gene intervention and cell supplementation, clear biological performance criteria for delivery systems have been established. However, the safe and efficient delivery to the degenerated intervertebral disc depends entirely on the suitable carrier. Therefore, it is clear that the rational design of delivery platforms for IVDD cannot be separated from accurate degeneration mechanisms, nor from a deep understanding of the manufacturing technologies and material properties that determine the carrier’s structure, mechanical properties, degradation rate, and payload release kinetics.

In summary, the molecular and cellular pathological mechanisms of IVDD clearly identify a series of intervention targets, including replenishing deficient cells, inhibiting catabolic enzymes, blocking inflammatory signals, and promoting matrix synthesis. However, the precise and efficient delivery of therapeutic cells or nucleic acids requires overcoming a series of delivery bottlenecks: (1) The harsh biochemical microenvironment requires carriers to protect bioactive molecules from rapid degradation or inactivation; (2) The dense ECM impedes carrier penetration and diffusion, requiring targeted and efficient cellular uptake strategies; (3) Intradiscal pressures as high as ~3 MPa require implants to possess sufficient mechanical integrity to prevent extrusion, while their mechanical properties must match the tissue modulus at different stages of degeneration to avoid stress shielding; (4) High rates of leakage post-injection are the primary cause of complications such as ectopic ossification, requiring delivery systems to possess rapid in situ cross-linking, tissue adhesion, or shape memory capabilities to ensure effective retention; (5) Gradually worsening nutrient deprivation during the degeneration process creates a supply-demand imbalance when introducing exogenous cells, requiring delivery systems to feature diffusion-enhancing porous structures or integrated metabolic support functions. These bottlenecks constitute the core evaluation criteria for the delivery systems. To develop a platform capable of addressing these multiple challenges, it is essential to understand the underlying manufacturing technologies and core materials. Therefore, in the following sections, we will outline the engineering techniques and material considerations for constructing IVDD delivery platforms, thereby providing a technical analytical framework for the subsequent evaluation of various cell- and gene-therapy carriers.

## 3. Engineering Fundamentals for IVD Delivery Systems

### 3.1. Fabrication Technologies for Delivery Systems

With clinical staging, pathophysiological mechanisms, and therapeutic gaps now established, we will now turn to the engineering principles that guide the delivery system design. The performance of cellular or gene carriers depends not only on their chemical components but also on the fabrication techniques that shape their structure, as well as the material properties that determine their mechanical behavior, degradation rates, and biocompatibility.

**Figure 3:**
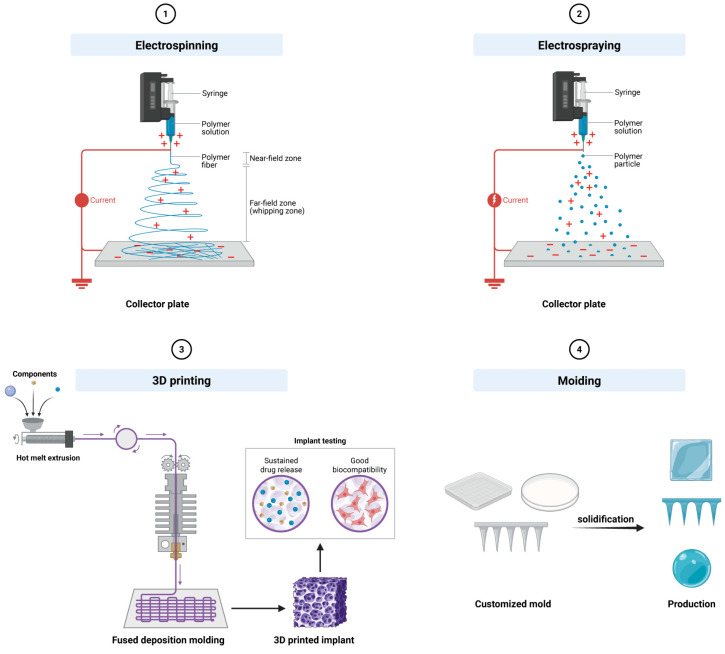
Schematic diagram of synthesis technologies commonly used in IVD delivery systems.

Four techniques dominate IVDD delivery system fabrication: electrospinning, electrospraying, additive manufacturing, and molding (Figure 3). Electrospinning uses a high-voltage field to draw polymer solutions into continuous nano- to micro-scale fibers collected as non-woven mats with a high surface-area-to-volume ratio and interconnected porosity [80,81]. By controlling fiber alignment and stacking sequence, electrospun scaffolds recapitulate the anisotropic tensile properties and hierarchical collagen architecture of native AF, making this the method of choice for AF repair where structural mimicry and mechanical competence are paramount [82,83]. Electrospraying employs a similar high-voltage setup but prevents continuous fiber formation, instead generating highly uniform micro- or nanoparticles ideal for encapsulating biological cargoes [84]. Because rapid solvent evaporation and gentle processing can preserve bioactivity [85], the resulting particles can be engineered with precise size distributions for controlled release kinetics and formulated as injectable suspensions. Additive manufacturing (3D printing) constructs patient-specific geometries layer by layer from digital models, enabling the spatially defined deposition of hydrogels via extrusion-based bioprinting [81,86]. Its principal advantage for IVDD lies in fabricating constructs with irregular, patient-matched shapes and structures [87,88].

Molding, the simplest approach, involves casting a polymer solution or hydrogel precursor into a cavity and solidifying by thermal gelation, chemical crosslinking, or photopolymerization. While lacking the spatial control of electrospinning or 3D printing, it is cost-effective, easily standardized, and well-suited for in vitro models and simple geometries, and can be combined with particulate leaching or freeze-drying to introduce porosity. This process shapes the material into specific forms tailored to the application [89]. Apart from these mainstream technologies, existing research has reported other fabrication strategies suitable for IVDD applications, including microfluidic techniques for microsphere fabrication [90], freeze-drying, and melt-based electrowriting [91,92].

Overall, the choice of fabrication technique must align with the therapeutic goal, target region, and required mechanical properties. An increasing number of studies are exploring hybrid methods that integrate multiple approaches to leverage complementary advantages.

### 3.2. Material Considerations for IVD Delivery Systems

The choice of polymer determines mechanical behavior, degradation rate, and biocompatibility, which must be matched to the target disc compartment and degeneration stage. Natural polymers such as hyaluronic acid (HA) and collagen provide intrinsic bioactivity but limited mechanical durability: unmodified HA hydrogels exhibit compressive moduli of 0.5–2 kPa and degrade within days to weeks, while collagen gels are even softer (0.1–1 kPa) and equally labile [93,94]. These values fall within the healthy NP range of approximately 0.5–5 kPa, making them suitable for early degeneration where the surrounding annulus fibrosus and endplates remain competent. However, more advanced degeneration may temporarily require a stiffer implant to restore disc height, provided stress shielding and adjacent segment disease are managed. Synthetic polymers offer tunable properties to overcome these mechanical and degradation limitations. Poly(lactic-co-glycolic acid) (PLGA) degrades over one to six months by adjusting the lactic-to-glycolic acid ratio [95,96], whereas polycaprolactone (PCL) persists for more than two years, conferring durable structural support for annulus fibrosus scaffolds but risking chronic foreign-body responses if not resorbed [97]. A common concern is the acidic degradation products of PLGA and polylactic acid (PLA), which lower local pH and damage encapsulated cells, thereby hindering the therapeutic process [98]. Additionally, premature degradation leads to disc height collapse and loss of therapeutic efficacy. An ideal system would degrade gradually, transferring mechanical loads to newly deposited tissue without sudden failure, and releasing only non-toxic byproducts. Another frequently overlooked consideration is the nutritional supply paradox. Degenerated intervertebral discs are nutritionally deprived; implanting metabolically active stem cells may accelerate necrosis, potentially exacerbating inflammation. This paradox requires delivery systems to possess diffusible porous structures, prioritize paracrine nutrition over structural replacement, or incorporate oxygen-producing materials and low cell seeding densities to integrate metabolic support. Furthermore, intradiscal pressure in human lumbar discs ranges from approximately 0.1–0.3 MPa at rest to 1.5–3.0 MPa during lifting, yet most hydrogel formulations have not been tested under such loads. Therefore, this critical gap must be addressed to ensure mechanical reliability in clinical translation.

Following the establishment of these principles of materials design, we can systematically evaluate their application in the two main treatment modalities for IVDD. For each therapy, we will examine how the engineered delivery platform directly links design architecture to the pathological challenges of degenerative intervertebral discs.

## 4. Platform-Driven Solutions to Core Delivery Challenges

The microenvironment of a degenerated intervertebral disc imposes stringent engineering requirements on any therapeutic delivery system. This section will analyze how to use this delivery system to overcome five key obstacles: mechanical loads that threaten structural integrity; high-pressure compression causing drug leakage; biological barriers impeding intracellular entry; demands for sustained drug release over weeks or months; and severe nutritional deficiencies leading to implant-related cellular malnutrition. A critical evaluation of the performance of different delivery systems is conducted based on existing quantitative benchmarks. This cross-platform direct comparison provides guidelines for selecting or designing drug delivery systems for IVDD (Table 1).

### 4.1. Mechanical Protection and Structural Integration

The IVD experiences complex multiaxial loading, with intradiscal pressures in human lumbar discs ranging from 0.1 to 3.0 MPa [4]. A delivery system must not only protect its encapsulated cargo from these forces but also either match the mechanical properties to avoid stress shielding or temporarily provide stronger support to restore disc height. Hydrogels are the platform of choice for mimicking the NP’s mechanical environment. Their high water content (90–95%) and viscoelastic properties approximate those of the native NP, which has a compressive modulus of 0.5–5 kPa [99,100,101]. Sakai et al. embedded MSCs in an Atelocollagen^®^ gel, which significantly increased glycosaminoglycan synthesis and delayed radiographic progression of the disease in a rabbit model [102]. As the primary glycosaminoglycan (GAG) component of the natural nucleus pulposus, hyaluronic acid has been widely used. Peroglio et al. grafted poly (N-isopropylacrylamide) onto an HA backbone to create an injectable thermoresponsive hydrogel that underwent physical crosslinking at 37 °C, significantly reducing viscosity at room temperature for ease of injection while providing a modulus sufficient for a contained NP [103]. However, most hydrogels possess compressive moduli below 10 kPa, which may be insufficient to resist extrusion under the elevated intradiscal pressures when the AF is fissured. 

In stark contrast, nanofiber scaffolds are uniquely designed to provide the anisotropic tensile strength required for annulus fibrosus repair [104]. These electrospun scaffolds can replicate the layered structure of the natural AF. Pirvu et al. developed a polymer scaffold seeded with human MSCs that upregulates the expression of type V collagen and anabolic genes [105]. Elsaadany et al. demonstrated that biphasic electrospun scaffolds can upregulate fibrocartilage marker gene expression by 5- to 30-fold under dynamic mechanical stimulation, indicating that mechanical signals transmitted through the scaffold structure can guide lineage-specific differentiation [106]. A major limitation of such scaffolds is their non-injectable nature, requiring surgical implantation. The trade-off between the injectability of hydrogels and their NP-like mechanical properties, on the one hand, and the high strength of nanofiber scaffolds and the need for surgical implantation, on the other, constitutes a fundamental design decision based on the target tissue site.

### 4.2. Efficient Retention and Leakage Prevention

Post-injection leakage is a primary failure mode for intradiscal therapies. Naked cell suspensions exhibit leakage rates of approximately 90%, and these escaped MSCs carry a documented risk of forming ectopic osteophytes [107]. A core function of any delivery vehicle is to transform the injectable cargo into a solid state to resist extrusion.

**Figure 4:**
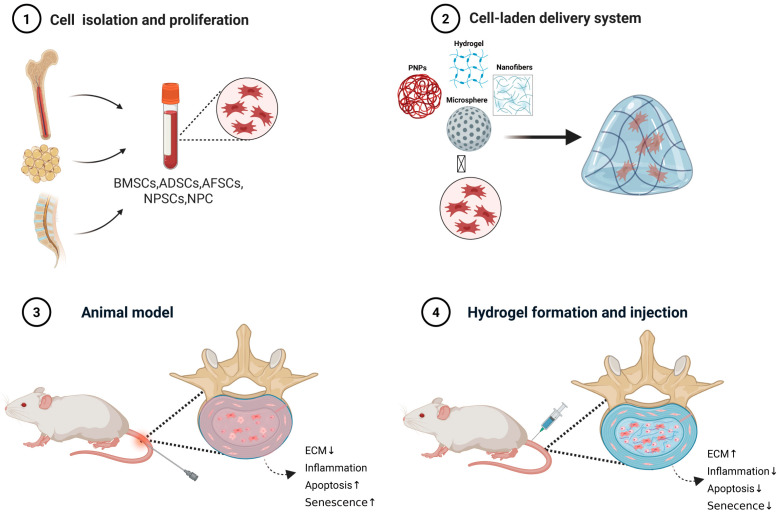
Common cell types and delivery systems used in cell therapy for IVDD (in hydrogel form, rat puncture model).

Hydrogels address this challenge through physical entrapment (Figure 4). Matrix-assisted cell transfer, in which cells are embedded in a hydrogel, reduces leakage from 90% to 50% [108]. However, this 50% leakage rate remains an unresolved issue, not a success. Other strategies are currently being developed to overcome this problem, such as introducing tissue-adhesive chemicals or designing systems that rapidly seal needle punctures. Ye et al. used a two-component polymer network hydrogel composed of GelMA and HAMA to encapsulate genetically engineered MSCs. This gene-cell composite hydrogel not only upregulated ECM synthesis but also provided a cohesive network structure, thereby limiting cell escape [109].

**Figure 5:**
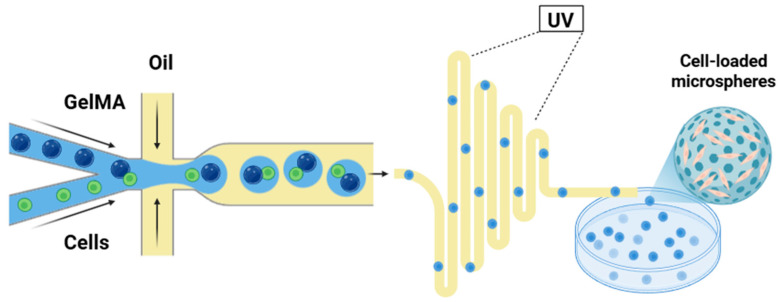
Microfluidic device for preparation of cell-loaded hydrogel microspheres.

Microspheres offer a more robust solution by encapsulating cells within discrete, solid particles to resist deformation and migration (Figure 5). Li et al. developed a collagen microencapsulation platform in which MSCs were encapsulated within solid microspheres composed of collagen nanofiber networks. In a six-month rabbit IVDD model, MSCs delivered in these microspheres markedly reduced the incidence of peridiscal bone formation compared to saline-delivered MSCs [110]. Nakielski et al. developed chitosan- and chondroitin sulfate-modified electrospun nanofiber microscaffolds seeded with BMSCs; the microscaffolds prevented cell leakage under high disc pressures when delivered via a minimally invasive approach in a porcine model [111]. The modular nature of microspheres allows for injection as a suspension, and their larger size relative to nanoparticles prolongs retention, directly tackling the hallmark complication of cell therapy. However, the long-term fragmentation behavior under cyclic mechanical loading remains inadequately characterized.

### 4.3. Biological Barrier Crossing and Targeted Intracellular Delivery

While cells require an extracellular niche, gene therapy vectors must overcome biological barriers such as the dense, negatively charged ECM, the cell membrane, endosomal entrapment, and the nuclear envelope. This challenge primarily falls to nanoparticles and viral/non-viral vectors, whose subcellular dimensions enable them to be engineered for efficient cellular uptake and intracellular trafficking.

Nanoparticles can exploit surface charge to passively target the negatively charged IVD matrix and can be modified for cell-specific uptake. Liang et al. constructed nanostructured PLGA particles loaded with dexamethasone and TGF-β3 and seeded with ADSCs; these particles generated more ECM than controls and achieved disc height recovery in a rat puncture model [98]. Ding et al. synthesized a reactive oxygen species (ROS)-responsive cationic copolymer, poly(β-amino ester)-poly(ε-caprolactone) (PBC), with high affinity for plasmid DNA compression and incorporated the anti-inflammatory flavonoid luteolin. This system inhibited the inflammatory response by activating TGF-β/SMAD3 and inhibiting NF-κB/p65 pathways, delaying IVDD in rats. Notwithstanding these outcomes, cytotoxicity remains a concern for many cationic PNPs, particularly those employing high-molecular-weight polycations that can disrupt cell membranes.

**Figure 6:**
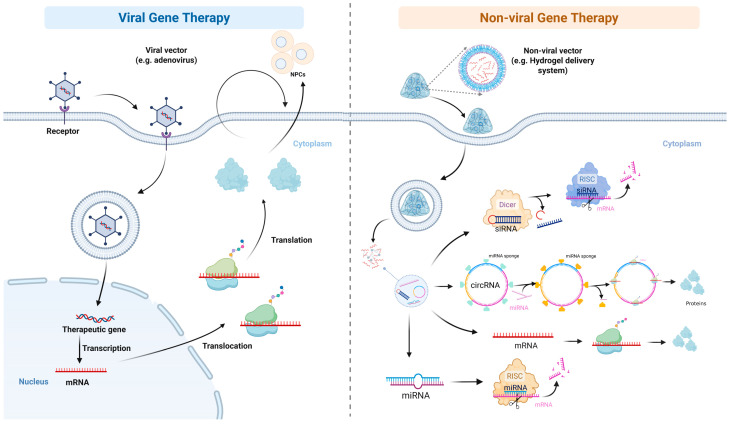
Schematic highlighting gene therapy for IVDD treatment. Viral/Non-viral vectors loaded the therapeutic genes into the cells, took effect in the cytoplasm/nucleus, and upregulated or downregulated the expression of the corresponding proteins.

Viral vectors represent the most efficient gene delivery systems (Figure 6), achieving transduction rates exceeding 80% in permissive cells [112]. Adenoviral vectors were the first explored and remain widely used. Zhang et al. systematically compared adenoviral transduction with various BMPs, finding that BMP-7 most potently stimulated proteoglycan production and BMP-2 most effectively promoted NPC proliferation [21,22,113]. Lattermann et al. demonstrated that AAV-mediated intradiscal gene transfer was feasible even in immunocompetent rabbits pre-exposed to the virus [114]. Mern et al. employed self-complementary AAV serotype 6 to knock down ADAMTS4 in human degenerative NPCs, achieving up to 87.7% knockdown sustained for at least 48 days [115]. The trade-off for this high efficiency is immunogenicity and potential genotoxicity. Levicoff et al. found that high-dose adenoviral injections caused bilateral lower limb paralysis in rabbits, underscoring that adverse effects depend on dose and delivery site, which must be carefully managed [116].

Non-viral vectors, such as cationic polymers and lipids, offer a safer profile and greater chemical flexibility, but suffer from substantially lower transfection efficiency (Figure 6). Furthermore, due to difficulties in penetrating the nuclei of non-proliferating NPCs, their expression is transient [117,118]. Srivastava et al. prepared a multifunctional gene vector based on cationic HA. This vector formed a complex with pDNA and successfully delivered it to bovine intervertebral disc cells, achieving excellent transfection efficiency and low cytotoxicity [119]. Feng et al. delivered heme oxygenase-1 (HO-1) pDNA into rat intervertebral discs via mixed cationic block copolymer micelles, significantly reducing the needling-induced inflammation and increasing GAG content [120]. Qingxin et al. developed a programmable DNA hydrogel loaded with miR-5590, providing a genetic microenvironment that triggered autophagy while inhibiting apoptosis through the PI3K/Akt/mTOR pathway [121]. Beyond this, recently emerging physical enhancement methods, such as electroporation, microbubble-enhanced ultrasound, and magnetically mediated transfection, have begun to narrow the efficiency gap between non-viral and viral systems [122,123].

### 4.4. Controlled Release Kinetics and Metabolic Support

Sustaining therapeutic effects in a chronic disease demands precise temporal control over therapeutic effects, while simultaneously addressing the nutrient-depleted environment to ensure the viability of the implanted cargo.

#### 4.4.1. Controlled Release and Expression Kinetics

Hydrogels and microspheres are leading platforms for controlling cargo release. Gan et al. fabricated a composite hydrogel with PLGA nanoparticles loaded with TGF-β3; the system provided sustained growth factor release and successfully induced MSC differentiation toward an NP-like phenotype with selective upregulation of ECM-related genes [124]. Ma et al. constructed a hybrid hydrogel from decellularized nucleus pulposus matrix and chitosan, combined with PLGA microspheres loaded with GDF-5; this system exhibited sustained-release behavior in vitro, promoting cell proliferation and differentiation, enhancing COL2A1 protein expression, thereby promoting NP tissue regeneration in a rat model [125].

In gene therapy, viral vectors provide transgene expression for months to years. Wang et al. employed lentiviral vectors, which integrate into the host genome, to deliver TGF-β3 to NPMSCs, generating a transgenic seed cell line that provided stable, long-term expression and attenuated IVDD when encapsulated in a GelMA/HAMA hydrogel [109]. Non-viral systems often require engineering for sustained release. Feng et al. developed hyperbranched polymer-based nano-multilamellar bodies with a “bivalve” structure for the in vivo transfection of pDNA encoding NR4A. Results confirmed that this delivery system persisted in the rat tail for over 30 days and alleviated pathological fibrosis in NP tissue [126]. Additionally, Chang et al. developed injectable circRNA-silencing hydrogel microspheres, a cationic liposome-based lipoplex targeting circSTC2 was grafted onto methacrylate-hyaluronic acid microspheres fabricated via microfluidics, thereby continuously promoting type II collagen and aggrecan synthesis in the nutrient-restricted microenvironment [127].

#### 4.4.2. Metabolic Support Within a Nutrient-Depleted Niche

The degenerated disc’s low glucose and oxygen levels directly threaten the survival of high-density cell implants. This requires delivery systems to function not only as carriers but also as metabolic support systems. Porosity is a key design feature. The interconnected porous architecture of nanofibrous scaffolds and some microspheres can facilitate nutrient diffusion from the endplates to the encapsulated cells, partially mitigating the risk of rapid necrotic core formation. For example, the robust and porous collagen microspheres developed by Li et al. supported MSC attachment, survival, proliferation, migration, differentiation, and matrix remodeling, likely by enabling better metabolite exchange [110]. Similarly, Tang et al. utilized microfluidic technology to synthesize a dual-network hydrogel microsphere (GelMA/HA-His-Mg^2+^). The Mg^2+^ component acts as a cell activator, enhancing cells’ ability to withstand adverse degenerative microenvironments; this microsphere system alleviated rat IVDD and promoted tissue regeneration [24]. The integration of oxygen-producing materials into hydrogels, prioritizing paracrine nutritional support for seeded cells, is also a direct response to this metabolic paradox. However, this key factor for intracellular-based therapy success is often overlooked. Despite the versatility of microsphere delivery systems, the advantages of modular co-delivery using microspheres also introduce complexity. First, the release kinetics of each encapsulated component must be considered, and the long-term fate of microsphere fragments in vivo has not yet been fully characterized. Moreover, microspheres injected as a suspension may precipitate or aggregate, leading to inconsistent distribution and resulting in variable therapeutic efficacy.

## 5. Translational Challenges

Despite substantial advances in preclinical research, translating drug delivery systems for IVDD from experimental models to clinical practice still faces major obstacles.

The overwhelming majority of in vivo efficacy data derive from rodent or rabbit models, whose spinal biomechanics, disc dimensions, and cellular turnover rates differ from those of humans. Rats and rabbits are quadrupedal, and their discs experience loading modes that only partially recapitulate the daily stress patterns of the human lumbar spine. Unfortunately, the disc pressures in these animals remain below human physiological limits [128]. Most hydrogels and microspheres tested in small animals have therefore not been evaluated under the loading conditions that would precipitate structural failure in a human patient. Furthermore, the diffusion distance from the endplate capillaries to the central NP scales with disc diameter, which ranges from approximately 2–4 mm in rodents to over 40 mm in the human lumbar spine [129]. Because diffusive transport time increases with the square of distance, nutrient and oxygen delivery to centrally implanted cells is a far more severe constraint in humans than in small animals. Thus, therapeutic strategies that appear effective in rodents may fail in the human disc due to nutrient starvation of the implanted cargo. Large animal models, such as sheep, goats, and pigs, whose intervertebral disc dimensions and spinal biomechanics are more closely approximated to the human condition, and their incorporation into the preclinical validation pipeline, together with standardized outcome measures, is essential to bridge the current gap between small-animal promise and clinical reality [129,130]. In addition, the follow-up duration used in the vast majority of IVDD delivery system studies is 4–8 weeks, which can capture early repair responses but is inadequate for assessing long-term safety endpoints, including ectopic bone formation, tumorigenesis, or late-stage implant extrusion. In a rabbit model, Vadala et al. reported that leakage of injected MSCs could induce osteophyte formation. This process would evolve over months and remain undetected within an 8-week observation period. The genotoxic risk associated with integrating viral vectors and the tumorigenic potential of residual undifferentiated cells in iPSC-derived products similarly require monitoring over time scales far exceeding current standards [131,132]. The issue similarly applies to material fatigue failure and re-extrusion of implanted structures under cyclic loading. Furthermore, these mechanical failures may occur long after the standard observation period has ended, potentially offsetting any early biological benefits. However, preclinical study protocols incorporating 6 to 12 months of continuous imaging and final histological examination remain rare.

Mechanical-biological paradoxes further complicate intradiscal drug delivery. The injection procedure itself imposes injury that may accelerate disc degeneration. Simultaneously, any residual puncture channel serves as a pathway for drug leakage. A prospective human study demonstrated that contrast injection through the AF accelerated subsequent radiographic degeneration, and needle puncture alone is sufficient to induce IVDD in rabbit and rat models [133,134]. Although delivery systems can reduce leakage compared with saline suspensions, the reduction achieved to date remains partial. Bertram et al. reported that matrix-assisted cell transfer lowered the fraction of leaked cells from approximately 90% to 50%, meaning that in every second injection, half of the administered cell dose still escapes the disc [108]. Leaking MSCs have been documented to produce osteophytes in animal models, and bioactive gene vectors that extravasate into the epidural space or foramina could elicit off-target effects [110]. The pedicle route has been proposed as a method to avoid puncturing the spinal canal. Still, the risk of intraspinal canal invasion and the possibility of substance diffusion along the endplate-vertebral body interface under high-pressure injection have not yet been fully characterized [135,136]. Therefore, the development of delivery systems capable of rapid in situ solidification to seal the needle site or matching the injection pressure of degenerated intervertebral discs to minimize backflow has become a top priority.

Metabolic constraints impose an additional layer of translational risk. The degenerated disc is avascular and nutritionally depleted, and oxygen tension can fall to approximately 1%; these levels may be insufficient to meet the metabolic demands of high-density stem cell implants [4]. Implanting metabolically active cells into this environment risks accelerating necrosis as the limited substrate is rapidly consumed. Therefore, delivery systems must address metabolic support to ensure that transplanted cells survive long enough to exert a therapeutic effect.

Finally, a prevailing lack of standardization in this field hinders cross-study comparisons and clinical translation. A wide variety of cell types are used, including BMSCs, ADSCs, synovial mesenchymal stem cells, NPDSCs, and cells derived from iPSCs. These possess distinct differentiation potentials and paracrine characteristics, and many studies do not report the minimum standards for MSC identification recommended by the International Society for Cell Therapy (ISCT) [137,138]. Doses range from 10^4^ to 10^7^ cells per intervertebral disc, and systematic dose–response evaluations are indeed lacking. Culture conditions vary widely, and these factors are known to modulate the phenotype and therapeutic efficacy of MSCs. Therefore, establishing consensus reporting standards that encompass cell identity, survival rate, expansion protocols, delivery methods, and at least one quantitative imaging result and one histological finding would significantly improve reproducibility and facilitate the compilation of evidence required for regulatory submissions. The establishment of an international registry, similar to those used in cartilage repair studies, dedicated to documenting IVDD cell and gene therapy research could further accelerate the transition to clinical applications.

## 6. Future Directions

### 6.1. New Approach Methodologies (NAMs) for Preclinical Screening

To improve the predictive value of preclinical evaluation and address the limitations of animal models mentioned above, New Approach Methodologies (NAMs) must be integrated into the delivery system development pipeline. By simulating the tensile, compressive, and bending loads encountered during daily human activities, these methods apply physiologically relevant cyclic loads to injectable hydrogels, microspheres, or composite structures in vitro. Such systems can systematically evaluate burst pressure thresholds, fatigue life, and extrusion risks before animal implantation, providing quantitative failure criteria that cannot be obtained from static in vitro culture or small animal studies. Complementing this, finite-element computational models parameterized with patient-specific imaging data can predict intradiscal stress distributions, nutrient concentration gradients, and degradation kinetics [139], thereby enabling in silico optimization of material properties and implantation parameters. The adoption of NAMs aligns with the principles of the 3Rs and improves the translational fidelity of preclinical data.

### 6.2. Endplate Preconditioning as a Combinatorial Strategy

Given that endplate calcification chokes off the nutrient supply to the disc and renders any cell- or gene-based therapy substantially less effective, a paradigm shift may be warranted in which therapeutic intervention begins with endplate preconditioning designed to restore permeability. Chemical decalcification using local delivery of calcium-chelating agents, such as EDTA or citric acid, could transiently reopen endplate porosity, thereby increasing the flux of glucose and oxygen into the central NP. Mechanical interventions, including low-intensity pulsed ultrasound and extracorporeal shock wave therapy, have been preliminarily explored to improve endplate permeability through microfracture remodeling and enhanced local blood flow. In a staged combinatorial protocol, endplate preconditioning would be performed first to restore nutrient access, followed days to weeks later by intradiscal injection of the regenerative delivery system into a disc, where the metabolic environment has been transiently improved. The safety and efficacy of this strategy must be rigorously validated in large-animal models that naturally develop endplate calcification.

### 6.3. Gene-Cell Combination Therapy

Gene therapy and cell therapy address complementary deficits. Gene therapy requires a sufficient population of endogenous target cells to transduce, yet advanced IVDD is characterized by severe cellular depletion. Cell therapy can replenish the cellular pool, but the transplanted cells must contend with the same harsh microenvironment. Delivering genetically engineered cells within a protective carrier system directly solves both problems. The delivered cells provide the cellular substrate for gene expression and simultaneous ECM production, while the therapeutic genes equip the cells with enhanced survival, anti-inflammatory, or anabolic capabilities. Representative studies include the lentiviral transduction of NPMSCs with TGF-β3 followed by encapsulation in GelMA/HAMA hydrogels, which demonstrated sustained growth factor secretion, attenuated IVDD, and accelerated ECM synthesis, and the co-encapsulation of p65 siRNA with NPCs in a multi-dynamic bond hydrogel, which combined gene silencing with cell supplementation to restore ECM homeostasis. Future systems may incorporate multiple therapeutic genes, multiple cell types, and smart biomaterials that respond to biochemical or mechanical cues to tailor therapeutic output.

From a regulatory perspective, integrated gene-cell-biomaterial systems are classified as combination medical products. It is critical to adopt a modular research paradigm in which separate safety evaluations are performed for each component before comprehensive combination tests, and clinically validated biomaterials are prioritized. This paradigm can accommodate the overlapping oversight of biologics, drugs, and medical device administrations and further streamline the progress of clinical transformation.

## 7. Conclusions

The intervertebral disc presents a uniquely challenging environment that demands a multi-faceted approach to delivery. This review has systematically dissected these demands into five core delivery challenges and demonstrated how diverse platforms are tailored to address them. By moving beyond isolated, material-centric thinking toward a challenge-oriented framework, the critical design trade-offs become clear: hydrogels for NP hydration and cell retention; microspheres are used to reduce cell leakage; and scaffolds provide compressive and tensile support at the implantation site; viral vectors for durable gene transfer, while non-viral vectors emphasize safety and delivery flexibility. Bridging the gap to clinical practice will require not incremental refinement but a concerted effort to solve the systemic translational barriers: the unresolved cell leakage rate, the nutrient-supply paradox, the predictive failures of small-animal models, and the deficit in standardization. The convergence of these efforts, linking fabrication science, materials engineering, and molecular biology within a challenge-oriented design paradigm, is the path toward realizing precision therapy for IVDD.

## Figures and Tables

**Table 1:** Comparative Summary of Delivery Platforms Organized by Core Delivery Challenge.

Core Challenge	Hydrogels	Microspheres	Nanoparticles	Nanofibrous Scaffolds	Viral Vectors	Non-Viral Vectors
Mechanical Support	Low modulus (0.5–5 kPa)	Negligible	Negligible	High tensile strength	Negligible	Negligible
Retention and Leakage Prevention	Reduce cell leakage through physical entrapment.	Solid particles resist deformation. Significantly reduces leakage.	Rapid clearance.	Non-injectable; Structural integrity prevents migration.	N/A	N/A
Targeted Intracellular Delivery	N/A	N/A	High; capable of surface-charge mediated targeting and cellular uptake.	N/A	High transduction efficiency; inherent tropism.	Lower transfection efficiency; chemically targetable.
Controlled Release	Diffusion/degradation-controlled release.	Tunable degradation for controlled release.	Sustained intracellular release.	Release from fiber coatings	Sustained transgene expression for months to years.	Transient expression; engineered for sustained release.
Metabolic Support	High water content; porous networks facilitate nutrient diffusion.	Porous microspheres can support cell infiltration and diffusion.	N/A	Interconnected porosity can be designed for nutrient flow.	N/A	N/A

## Data Availability

No original data were produced in this study.

## Abbreviations

AAV	adeno-associated virus
ADAMTS	a disintegrin and metalloproteinase with thrombospondin motifs
AF	annulus fibrosus
BMP	bone morphogenetic protein
ECM	extracellular matrix
GDF-5	growth differentiation factor 5
HA	hyaluronic acid
IL-1Ra	interleukin-1 receptor antagonist
iPSC	induced pluripotent stem cell
IVD	intervertebral disk
IVDD	intervertebral disk degeneration
LBP	low back pain
MMP	matrix metalloproteinase
MSC	mesenchymal stem cell
NAMs	new approach methodologies
NF-κB	nuclear factor kappa-light-chain-enhancer of activated B cells
NP	nucleus pulposus
NPC	nucleus pulposus cell
NPMSC	nucleus pulposus mesenchymal stem cell
NSAIDs	nonsteroidal anti-inflammatory drugs
PCL	polycaprolactone
pDNA	plasmid DNA
PLGA	poly(lactic-co-glycolic acid)
ROS	reactive oxygen species
siRNA	small interfering RNA
TGF-β3	transforming growth factor beta 3
TIMP	tissue inhibitor of metalloproteinases

## References

[1] Maher C., Underwood M., Buchbinder R. (2017). Non-specific low back pain. Lancet.

[2] Chou R. (2021). Low Back Pain. Ann. Intern. Med..

[3] Mohd Isa I.L., Teoh S.L., Mohd Nor N.H., Mokhtar S.A. (2022). Discogenic Low Back Pain: Anatomy, Pathophysiology and Treatments of Intervertebral Disc Degeneration. Int. J. Mol. Sci..

[4] Hammoor B.T., Lai C.S., Xiong G.X., Elliott D.M., Snyder B., Vresilovic E., Bono C.M., Freedman B.R. (2026). Intervertebral disc degeneration. Nat. Rev. Dis. Prim..

[5] Kabeer A.S., Osmani H.T., Patel J., Robinson P., Ahmed N. (2023). The adult with low back pain: Causes, diagnosis, imaging features and management. Br. J. Hosp. Med..

[6] Fraser R.D., Osti O.L., Vernon-Roberts B. (1993). Intervertebral disc degeneration. Eur. Spine J..

[7] Roh E.J., Darai A., Kyung J.W., Choi H., Kwon S.Y., Bhujel B., Kim K.T., Han I. (2021). Genetic Therapy for Intervertebral Disc Degeneration. Int. J. Mol. Sci..

[8] Krut Z., Pelled G., Gazit D., Gazit Z. (2021). Stem Cells and Exosomes: New Therapies for Intervertebral Disc Degeneration. Cells.

[9] Khan A.N., Jacobsen H.E., Khan J., Filippi C.G., Levine M., Lehman R.A., Riew K.D., Lenke L.G., Chahine N.O. (2017). Inflammatory biomarkers of low back pain and disc degeneration: A review. Ann. N. Y. Acad. Sci..

[10] Chen X., Zhang A., Zhao K., Gao H., Shi P., Chen Y., Cheng Z., Zhou W., Zhang Y. (2024). The role of oxidative stress in intervertebral disc degeneration: Mechanisms and therapeutic implications. Ageing Res. Rev..

[11] Taylor W., Erwin W.M. (2024). Intervertebral Disc Degeneration and Regeneration: New Molecular Mechanisms and Therapeutics: Obstacles and Potential Breakthrough Technologies. Cells.

[12] Li Y., Zhang Y., Wang S., Ma X., Dai C., Wang Y., Ye C., Pan S., Gao C., Li W. (2025). Synergistic reversal of inflammation-mediated degeneration in intervertebral discs: Phenylboric acid-grafted hyaluronic acid hydrogel as an anti-oxidative vehicle for Timp-3 delivery and promotion of extracellular matrix synthesis. Acta Biomater..

[13] Ngo K., Pohl P., Wang D., Leme A.S., Lee J., Di P., Roughley P., Robbins P.D., Niedernhofer L.J., Sowa G. (2017). ADAMTS5 Deficiency Protects Mice From Chronic Tobacco Smoking-induced Intervertebral Disc Degeneration. Spine.

[14] Li Q., Guo R., Wu Z., Zhao C., Chen X., Wang H., Shen C. (2024). Endplate chondrocyte-derived exosomal miR-128-3p mitigates intervertebral disc degeneration by targeting TRAF6 via the miR-128-3p/TRAF6 axis to suppress pyroptosis. Int. Immunopharmacol..

[15] Dong W., Liu J., Lv Y., Wang F., Liu T., Sun S., Liao B., Shu Z., Qian J. (2019). miR-640 aggravates intervertebral disc degeneration via NF-κB and WNT signalling pathway. Cell Prolif..

[16] Hong Y., Duan Y., Zhu Z., Yu Q., Mo Z., Wang H., Zhou T., Liu Z., Bai J., Zhang X. (2024). IL-1ra loaded chondroitin sulfate-functionalized microspheres for minimally invasive treatment of intervertebral disc degeneration. Acta Biomater..

[17] Xia J., Zhang W., Jiang Y., Li T., Dong H., Zhu L., Xia Q., Zhao Y., Yi J., Weng Z. (2026). HACE1 alleviates intervertebral disc degeneration by inhibiting ferroptosis in nucleus pulposus cells. Sci. Rep..

[18] Zhang L., Cong X., Sun S., Li P., Zhao W., Wang N., Shen D., Zhang Y. (2026). Targeting EGFR with miR-148a-3p: A novel approach to mitigate intervertebral disc degeneration. J. Neurosurg. Spine.

[19] Wu Z.L., Ran R., Xie Q.Q., Zhang C., Chen Y.J., Cheng P., Wang K.P., Zhang H.H. (2026). FGF21-Mediated Upregulation of SIRT1 Delays Intervertebral Disc Degeneration by Promoting PINK1/Parkin Dependent Mitophagy Through Deacetylation of FOXO3. Aging Cell.

[20] Jiang C., Guo Q., Jin Y., Xu J.J., Sun Z.M., Zhu D.C., Lin J.H., Tian N.F., Sun L.J., Zhang X.L. (2019). Inhibition of EZH2 ameliorates cartilage endplate degeneration and attenuates the progression of intervertebral disc degeneration via demethylation of Sox-9. EBioMedicine.

[21] Zhang Y., Markova D., Im H.J., Hu W., Thonar E.J., He T.C., An H.S., Phillips F.M., Anderson D.G. (2009). Primary bovine intervertebral disc cells transduced with adenovirus overexpressing 12 BMPs and Sox9 maintain appropriate phenotype. Am. J. Phys. Med. Rehabil..

[22] Zhang Y., Phillips F.M., Thonar E.J., Oegema T., An H.S., Roman-Blas J.A., He T.C., Anderson D.G. (2008). Cell therapy using articular chondrocytes overexpressing BMP-7 or BMP-10 in a rabbit disc organ culture model. Spine.

[23] Sakai D., Mochida J., Yamamoto Y., Nomura T., Okuma M., Nishimura K., Nakai T., Ando K., Hotta T. (2003). Transplantation of mesenchymal stem cells embedded in Atelocollagen gel to the intervertebral disc: A potential therapeutic model for disc degeneration. Biomaterials.

[24] Tang Y., Zhang K., Zhou H., Zhang C., Liu Z., Chen H., Li H., Chen K. (2023). Transplantation of active nucleus pulposus cells with a keep-charging hydrogel microsphere system to rescue intervertebral disc degeneration. J. Nanobiotechnol..

[25] Xu P., Lou L., Zhan W., Wang C., Wu S., Liu Z., Wang Y. (2024). Bicomponent hydrogel laden with TGF-β3-nucleus pulposus stem cells for disc degeneration repair. Chem. Eng. J..

[26] Kadow T., Sowa G., Vo N., Kang J.D. (2015). Molecular basis of intervertebral disc degeneration and herniations: What are the important translational questions?. Clin. Orthop. Relat. Res..

[27] Ke W., Xu H., Zhang C., Liao Z., Liang H., Tong B., Yuan F., Wang K., Hua W., Wang B. (2025). An overview of mechanical microenvironment and mechanotransduction in intervertebral disc degeneration. Exp. Mol. Med..

[28] Ząbek Z., Wyczałkowska-Tomasik A., Poboży K., Adamus J.P., Turek G., Ząbek M., Pączek L. (2025). Understanding the Microenvironment of Intervertebral Disc Degeneration: A Comprehensive Review of Pathophysiological Insights and Therapeutic Implications. Int. J. Mol. Sci..

[29] Grunhagen T., Shirazi-Adl A., Fairbank J.C.T., Urban J.P.G. (2011). Intervertebral Disk Nutrition: A Review of Factors Influencing Concentrations of Nutrients and Metabolites. Orthop. Clin. N. Am..

[30] Vadalà G., Sowa G., Hubert M., Gilbertson L.G., Denaro V., Kang J.D. (2012). Mesenchymal stem cells injection in degenerated intervertebral disc: Cell leakage may induce osteophyte formation. J. Tissue Eng. Regen. Med..

[31] Chen Y., Zhang L., Shi X., Han J., Chen J., Zhang X., Xie D., Li Z., Niu X., Chen L. (2024). Characterization of the Nucleus Pulposus Progenitor Cells via Spatial Transcriptomics. Adv. Sci..

[32] Zhang A., Cheng Z., Chen Y., Shi P., Gan W., Zhang Y. (2023). Emerging tissue engineering strategies for annulus fibrosus therapy. Acta Biomater..

[33] Yang S., Zhang F., Ma J., Ding W. (2020). Intervertebral disc ageing and degeneration: The antiapoptotic effect of oestrogen. Ageing Res. Rev..

[34] Mohd Isa I.L., Mokhtar S.A., Abbah S.A., Fauzi M.B., Devitt A., Pandit A. (2022). Intervertebral Disc Degeneration: Biomaterials and Tissue Engineering Strategies toward Precision Medicine. Adv. Healthc. Mater..

[35] Dowdell J., Erwin M., Choma T., Vaccaro A., Iatridis J., Cho S.K. (2017). Intervertebral Disk Degeneration and Repair. Neurosurgery.

[36] Liu Y., Yang J., Wang Z., Fu J., Liu H., Lu Z., Dong J., Li Z., Mao Q., Li C. (2025). Lactate and lactylation in intervertebral disc degeneration. Front. Mol. Biosci..

[37] Zhang T., Feng P., Alexander P.G., Lee J.Y., Sowa G.A., Vo N.V. (2026). Lactate Metabolism in the Intervertebral Disc: Mechanistic Insights and Pathological Implications. Biomolecules.

[38] Ashinsky B., Smith H.E., Mauck R.L., Gullbrand S.E. (2021). Intervertebral disc degeneration and regeneration: A motion segment perspective. Eur. Cells Mater..

[39] Wang J., Zhang Y., Cao J., Wang Y., Anwar N., Zhang Z., Zhang D., Ma Y., Xiao Y., Xiao L. (2023). The role of autophagy in bone metabolism and clinical significance. Autophagy.

[40] Zeng Y., Danielson K.G., Albert T.J., Shapiro I.M., Risbud M.V. (2007). HIF-1 alpha is a regulator of galectin-3 expression in the intervertebral disc. J. Bone Miner. Res. Off. J. Am. Soc. Bone Miner. Res..

[41] Risbud M.V., Albert T.J., Guttapalli A., Vresilovic E.J., Hillibrand A.S., Vaccaro A.R., Shapiro I.M. (2004). Differentiation of Mesenchymal Stem Cells Towards a Nucleus Pulposus-like Phenotype In Vitro: Implications for Cell-Based Transplantation Therapy. Spine.

[42] Gruber H.E., Hanley E.N. (2003). Recent advances in disc cell biology. Spine.

[43] Le Maitre C.L., Freemont A.J., Hoyland J.A. (2007). Accelerated cellular senescence in degenerate intervertebral discs: A possible role in the pathogenesis of intervertebral disc degeneration. Arthritis Res. Ther..

[44] Gruber H.E., Ingram J.A., Davis D.E., Hanley E.N. (2009). Increased cell senescence is associated with decreased cell proliferation in vivo in the degenerating human annulus. Spine J. Off. J. N. Am. Spine Soc..

[45] Smith L.J., Nerurkar N.L., Choi K.S., Harfe B.D., Elliott D.M. (2011). Degeneration and regeneration of the intervertebral disc: Lessons from development. Dis. Model. Mech..

[46] Wu W., Cheng Z., Chen X., Shi P., Zhang A., Gao H., Wu W., Zhang Y. (2026). Pyroptosis: Mechanism and therapeutic strategies with intervertebral disc degeneration. Exp. Mol. Med..

[47] Risbud M.V., Shapiro I.M. (2014). Role of cytokines in intervertebral disc degeneration: Pain and disc content. Nat. Rev. Rheumatol..

[48] Cheng Z., Xiang Q., Wang J., Zhang Y. (2021). The potential role of melatonin in retarding intervertebral disc ageing and degeneration: A systematic review. Ageing Res. Rev..

[49] Sun K., Jing X., Guo J., Yao X., Guo F. (2021). Mitophagy in degenerative joint diseases. Autophagy.

[50] Lee J.Y., Ernestus R.I., Schröder R., Klug N. (2000). Histological study of lumbar intervertebral disc herniation in adolescents. Acta Neurochir..

[51] van Eerd M., Patijn J., Loeffen D., van Kleef M., Wildberger J. (2021). The Diagnostic Value of an X-ray-based Scoring System for Degeneration of the Cervical Spine: A Reproducibility and Validation Study. Pain Pract. Off. J. World Inst. Pain.

[52] Senck S., Trieb K., Kastner J., Hofstaetter S.G., Lugmayr H., Windisch G. (2020). Visualization of intervertebral disc degeneration in a cadaveric human lumbar spine using microcomputed tomography. J. Anat..

[53] Modic M.T., Steinberg P.M., Ross J.S., Masaryk T.J., Carter J.R. (1988). Degenerative disk disease: Assessment of changes in vertebral body marrow with MR imaging. Radiology.

[54] Takashima H., Yoshimoto M., Ogon I., Takebayashi T., Imamura R., Akatsuka Y., Yamashita T. (2023). T1rho, T2, and T2* relaxation time based on grading of intervertebral disc degeneration. Acta Radiol..

[55] Wang Y.X.J. (2022). Several concerns on grading lumbar disc degeneration on MR image with Pfirrmann criteria. J. Orthop. Transl..

[56] Pfirrmann C.W., Metzdorf A., Zanetti M., Hodler J., Boos N. (2001). Magnetic resonance classification of lumbar intervertebral disc degeneration. Spine.

[57] Urrutia J., Besa P., Campos M., Cikutovic P., Cabezon M., Molina M., Cruz J.P. (2016). The Pfirrmann classification of lumbar intervertebral disc degeneration: An independent inter- and intra-observer agreement assessment. Eur. Spine J..

[58] Dudli S., Fields A.J., Samartzis D., Karppinen J., Lotz J.C. (2016). Pathobiology of Modic changes. Eur. Spine J..

[59] Raj P.P. (2008). Intervertebral disc: Anatomy-physiology-pathophysiology-treatment. Pain Pract. Off. J. World Inst. Pain.

[60] Dang L., Liu Z. (2010). A review of current treatment for lumbar disc herniation in children and adolescents. Eur. Spine J..

[61] Kamali A., Ziadlou R., Lang G., Pfannkuche J., Cui S., Li Z., Richards R.G., Alini M., Grad S. (2021). Small molecule-based treatment approaches for intervertebral disc degeneration: Current options and future directions. Theranostics.

[62] Hoeberechts P.M. (1955). Surgical technique in herniated intervertebral disc. Can. Med. Assoc. J..

[63] Xu S., Liang Y., Zhu Z., Qian Y., Liu H. (2018). Adjacent segment degeneration or disease after cervical total disc replacement: A meta-analysis of randomized controlled trials. J. Orthop. Surg. Res..

[64] Yamagishi A., Sakaura H., Ishii M., Ohnishi A., Ohwada T. (2021). Postoperative Loss of Lumbar Lordosis Affects Clinical Outcomes in Patients with Pseudoarthrosis after Posterior Lumbar Interbody Fusion Using Cortical Bone Trajectory Screw Fixation. Asian Spine J..

[65] Ilic M.Z., East C.J., Rogerson F.M., Fosang A.J., Handley C.J. (2007). Distinguishing aggrecan loss from aggrecan proteolysis in ADAMTS-4 and ADAMTS-5 single and double deficient mice. J. Biol. Chem..

[66] Bayliss M.T., Hutton S., Hayward J., Maciewicz R.A. (2001). Distribution of aggrecanase (ADAMts 4/5) cleavage products in normal and osteoarthritic human articular cartilage: The influence of age, topography and zone of tissue. Osteoarthr. Cartil..

[67] Gu S.X., Li X., Hamilton J.L., Chee A., Kc R., Chen D., An H.S., Kim J.S., Oh C.D., Ma Y.Z. (2015). MicroRNA-146a reduces IL-1 dependent inflammatory responses in the intervertebral disc. Gene.

[68] Olivieri F., Prattichizzo F., Giuliani A., Matacchione G., Rippo M.R., Sabbatinelli J., Bonafè M. (2021). miR-21 and miR-146a: The microRNAs of inflammaging and age-related diseases. Ageing Res. Rev..

[69] Hua T., Yang M., Song H., Kong E., Deng M., Li Y., Li J., Liu Z., Fu H., Wang Y. (2022). Huc-MSCs-derived exosomes attenuate inflammatory pain by regulating microglia pyroptosis and autophagy via the miR-146a-5p/TRAF6 axis. J. Nanobiotechnol..

[70] Ding J., Zhang Y., Cai X., Zhang Y., Yan S., Wang J., Zhang S., Yin T., Yang C., Yang J. (2021). Extracellular vesicles derived from M1 macrophages deliver miR-146a-5p and miR-146b-5p to suppress trophoblast migration and invasion by targeting TRAF6 in recurrent spontaneous abortion. Theranostics.

[71] Lawrence T. (2009). The nuclear factor NF-kappaB pathway in inflammation. Cold Spring Harb. Perspect. Biol..

[72] Guo S., Cui L., Xiao C., Wang C., Zhu B., Liu X., Li Y., Liu X., Wang D., Li S. (2021). The Mechanisms and Functions of GDF-5 in Intervertebral Disc Degeneration. Orthop. Surg..

[73] Khalid S., Ekram S., Ramzan F., Salim A., Khan I. (2023). Co-regulation of Sox9 and TGFβ1 transcription factors in mesenchymal stem cells regenerated the intervertebral disc degeneration. Front. Med..

[74] Lee S., Yu Y., Kim D.H., Bock M., Kim Y., An S.B., Choi H., Shin H.E., Hwang D.Y., Han I. (2025). Enhanced disc regeneration through CRISPR/Cas9-mediated SOX9 and TGFβ1 coexpression in tonsil-derived mesenchymal stromal cells. Stem Cell Res. Ther..

[75] Yoon S.T., Park J.S., Kim K.S., Li J., Attallah-Wasif E.S., Hutton W.C., Boden S.D. (2004). ISSLS prize winner: LMP-1 upregulates intervertebral disc cell production of proteoglycans and BMPs in vitro and in vivo. Spine.

[76] Sakai D., Andersson G.B. (2015). Stem cell therapy for intervertebral disc regeneration: Obstacles and solutions. Nat. Rev. Rheumatol..

[77] Ankrum J.A., Ong J.F., Karp J.M. (2014). Mesenchymal stem cells: Immune evasive, not immune privileged. Nat. Biotechnol..

[78] Miyamoto T., Muneta T., Tabuchi T., Matsumoto K., Saito H., Tsuji K., Sekiya I. (2010). Intradiscal transplantation of synovial mesenchymal stem cells prevents intervertebral disc degeneration through suppression of matrix metalloproteinase-related genes in nucleus pulposus cells in rabbits. Arthritis Res. Ther..

[79] Barcena A.J.R., Perez J.V.D., Bernardino M.R., Damasco J.A., San Valentin E.M.D., Klusman C., Martin B., Canlas G.M., Heralde F.M., Fowlkes N. (2025). Bismuth-infused perivascular wrap facilitates delivery of mesenchymal stem cells and attenuation of neointimal hyperplasia in rat arteriovenous fistulas. Biomater. Adv..

[80] Barcena A.J.R., Perez J.V.D., Damasco J.A., Bernardino M.R., San Valentin E.M.D., Klusman C., Martin B., Cortes A., Canlas G.M., Del Mundo H.C. (2023). Gold Nanoparticles for Monitoring of Mesenchymal Stem-Cell-Loaded Bioresorbable Polymeric Wraps for Arteriovenous Fistula Maturation. Int. J. Mol. Sci..

[81] De Pieri A., Byerley A.M., Musumeci C.R., Salemizadehparizi F., Vanderhorst M.A., Wuertz-Kozak K. (2020). Electrospinning and 3D bioprinting for intervertebral disc tissue engineering. JOR Spine.

[82] Lazebnik M., Singh M., Glatt P., Friis L.A., Berkland C.J., Detamore M.S. (2011). Biomimetic method for combining the nucleus pulposus and annulus fibrosus for intervertebral disc tissue engineering. J. Tissue Eng. Regen. Med..

[83] Tu Z., Han F., Zhu Z., Yu Q., Liu C., Bao Y., Li B., Zhou F. (2023). Sustained release of basic fibroblast growth factor in micro/nanofibrous scaffolds promotes annulus fibrosus regeneration. Acta Biomater..

[84] Loepfe M., Duss A., Zafeiropoulou K.A., Björgvinsdóttir O., D’Este M., Eglin D., Fortunato G., Klasen J., Ferguson S.J., Wuertz-Kozak K. (2019). Electrospray-Based Microencapsulation of Epigallocatechin 3-Gallate for Local Delivery into the Intervertebral Disc. Pharmaceutics.

[85] Xu H., Sun M., Wang C., Xia K., Xiao S., Wang Y., Ying L., Yu C., Yang Q., He Y. (2020). Growth differentiation factor-5-gelatin methacryloyl injectable microspheres laden with adipose-derived stem cells for repair of disc degeneration. Biofabrication.

[86] Astudillo Potes M.D., Tilton M., Mitra I., Liu X., Dashtdar B., Camilleri E.T., Elder B.D., Lu L. (2025). 3D Bioprinted Chondrogenic Gelatin Methacrylate-Poly(ethylene glycol) Diacrylate Composite Scaffolds for Intervertebral Disc Restoration. Int. J. Extrem. Manuf..

[87] Liu Z., Wang H., Yuan Z., Wei Q., Han F., Chen S., Xu H., Li J., Wang J., Li Z. (2022). High-resolution 3D printing of angle-ply annulus fibrosus scaffolds for intervertebral disc regeneration. Biofabrication.

[88] Sha Z., Ma Y., Li W., Cao Y., He Z., Zheng L., Lv H., Zhou X., He C., Zhu R. (2025). 3D-Printed Negative Poisson’s Ratio Composite Scaffolds with Antibacterial and Microenvironment Remodeling Capacities for Ameliorating Intervertebral Disc Degeneration. Adv. Healthc. Mater..

[89] Meng Q., Xie E., Sun H., Wang H., Li J., Liu Z., Li K., Hu J., Chen Q., Liu C. (2024). High-Strength Smart Microneedles with “Offensive and Defensive” Effects for Intervertebral Disc Repair. Adv. Mater..

[90] Chen Y., Yang Z.R., Cheng Z., Shi P., Zhang A., Fan J.W., Zhao Z., Jiang H., Zhu J., Zhang Y. (2025). Injectable hydrogel microspheres promoting inflammation modulation and nucleus pulposus-like differentiation for intervertebral disc regeneration. J. Control. Release.

[91] Nie M.D., Bao B.K., Zhang N.Z., Cheng R.S., Fu L.J., Cheng C.K. (2025). Dumbbell-shaped hydrogel plug for annulus fibrosus repair: From material design to in vivo validation. J. Orthop. Transl..

[92] Carrot E., Chaaban M., Contreras D.C., Schiex C., Véziers J., Halgand B., Loll F., Clouet J., Monaghan M.G., Fusellier M. (2025). Biofabrication of an ovine intervertebral disc model by combining a polycaprolactone frame with a bioprinted alginate hydrogel. Biofabrication.

[93] Li Z., Kaplan K.M., Wertzel A., Peroglio M., Amit B., Alini M., Grad S., Yayon A. (2014). Biomimetic fibrin-hyaluronan hydrogels for nucleus pulposus regeneration. Regen. Med..

[94] Russo F., Ambrosio L., Peroglio M., Guo W., Wangler S., Gewiess J., Grad S., Alini M., Papalia R., Vadalà G. (2021). A Hyaluronan and Platelet-Rich Plasma Hydrogel for Mesenchymal Stem Cell Delivery in the Intervertebral Disc: An Organ Culture Study. Int. J. Mol. Sci..

[95] Zhang T., Wang Y., Li R., Xin J., Zheng Z., Zhang X., Xiao C., Zhang S. (2023). ROS-responsive magnesium-containing microspheres for antioxidative treatment of intervertebral disc degeneration. Acta Biomater..

[96] Lim S., An S.B., Jung M., Joshi H.P., Kumar H., Kim C., Song S.Y., Lee J.R., Kang M., Han I. (2022). Local Delivery of Senolytic Drug Inhibits Intervertebral Disc Degeneration and Restores Intervertebral Disc Structure. Adv. Healthc. Mater..

[97] Wang Y., Zheng G., Xie X., Yu W., Wang J., Zang F., Yang C., Xiao Q., Zhang R., Wei L. (2023). Low-dose celecoxib-loaded PCL fibers reverse intervertebral disc degeneration by up-regulating CHSY3 expression. J. Nanobiotechnol..

[98] Liang C.Z., Li H., Tao Y.Q., Peng L.H., Gao J.Q., Wu J.J., Li F.C., Hua J.M., Chen Q.X. (2013). Dual release of dexamethasone and TGF-β3 from polymeric microspheres for stem cell matrix accumulation in a rat disc degeneration model. Acta Biomater..

[99] Zhang J., Li C., Liu H., Wang Q., Xu A.Y., Shah K., Qi L., Wang Z., Zhang X., Huang C. (2025). Hydrogel delivery systems in intervertebral disc degeneration: Current status and future perspectives. J. Control. Release.

[100] Wu W., Cheng Z., Shi P., Gao H., Chen X., Wu W., Yu Z., Yang C., Zhang Y. (2026). Microenvironment-educated MSC-EVs loaded injectable smart hydrogel for targeting senescent nucleus pulposus cells and inhibiting ferroptosis against intervertebral disc degeneration. Bioact. Mater..

[101] Wu W., Cheng Z., Shi P., Gao H., Chen Y., Zhang A., Chen X., Wu W., Zhang Y. (2026). Ultrasound-responsive piezoelectric hydrogel attenuates oxidative stress-induced nucleus pulposus senescence via AMPK-FOXO1a-mediated mitophagy for intervertebral disc regeneration. Biomaterials.

[102] Colella F., Garcia J.P., Sorbona M., Lolli A., Antunes B., D’Atri D., Barré F.P.Y., Oieni J., Vainieri M.L., Zerrillo L. (2020). Drug delivery in intervertebral disc degeneration and osteoarthritis: Selecting the optimal platform for the delivery of disease-modifying agents. J. Control. Release.

[103] Jiang Y., Wang J., Sun D., Liu Z., Qi L., Du M., Wang J., Li Y., Zhu C., Huang Y. (2023). A hydrogel reservoir as a self-contained nucleus pulposus cell delivery vehicle for immunoregulation and repair of degenerated intervertebral disc. Acta Biomater..

[104] Li C., Chen J., Lv Y., Liu Y., Guo Q., Wang J., Wang C., Hu P., Liu Y. (2022). Recent Progress in Electrospun Nanofiber-Based Degenerated Intervertebral Disc Repair. ACS Biomater. Sci. Eng..

[105] Pirvu T., Blanquer S.B., Benneker L.M., Grijpma D.W., Richards R.G., Alini M., Eglin D., Grad S., Li Z. (2015). A combined biomaterial and cellular approach for annulus fibrosus rupture repair. Biomaterials.

[106] Elsaadany M., Winters K., Adams S., Stasuk A., Ayan H., Yildirim-Ayan E. (2017). Equiaxial Strain Modulates Adipose-derived Stem Cell Differentiation within 3D Biphasic Scaffolds towards Annulus Fibrosus. Sci. Rep..

[107] Cao G., Zhang S., Wang Y., Quan S., Yue C., Yao J., Alexander P.G., Tan H. (2023). Pathogenesis of acquired heterotopic ossification: Risk factors, cellular mechanisms, and therapeutic implications. Bone.

[108] Bertram H., Kroeber M., Wang H., Unglaub F., Guehring T., Carstens C., Richter W. (2005). Matrix-assisted cell transfer for intervertebral disc cell therapy. Biochem. Biophys. Res. Commun..

[109] Ye Y., Xu P., Li C., Jin S., Hu J., Fang Y., Zhu K., Xu G., Han Z., Zhang Z. (2023). Bioactive hydrogel encapsulated dual-gene engineered nucleus pulposus stem cells towards intervertebral disc tissue repair. Chem. Eng. J..

[110] Li Y.Y., Diao H.J., Chik T.K., Chow C.T., An X.M., Leung V., Cheung K.M., Chan B.P. (2014). Delivering mesenchymal stem cells in collagen microsphere carriers to rabbit degenerative disc: Reduced risk of osteophyte formation. Tissue Eng. Part A.

[111] Nakielski P., Rybak D., Jezierska-Woźniak K., Rinoldi C., Sinderewicz E., Staszkiewicz-Chodor J., Haghighat Bayan M.A., Czelejewska W., Urbanek O., Kosik-Kozioł A. (2023). Minimally Invasive Intradiscal Delivery of BM-MSCs via Fibrous Microscaffold Carriers. ACS Appl. Mater. Interfaces.

[112] Vadalà G., Sowa G.A., Kang J.D. (2007). Gene therapy for disc degeneration. Expert Opin. Biol. Ther..

[113] Zhang Y., Li Z., Thonar E.J., An H.S., He T.C., Pietryla D., Phillips F.M. (2005). Transduced bovine articular chondrocytes affect the metabolism of cocultured nucleus pulposus cells in vitro: Implications for chondrocyte transplantation into the intervertebral disc. Spine.

[114] Lattermann C., Oxner W.M., Xiao X., Li J., Gilbertson L.G., Robbins P.D., Kang J.D. (2005). The adeno associated viral vector as a strategy for intradiscal gene transfer in immune competent and pre-exposed rabbits. Spine.

[115] Mern D.S., Tschugg A., Hartmann S., Thomé C. (2017). Self-complementary adeno-associated virus serotype 6 mediated knockdown of ADAMTS4 induces long-term and effective enhancement of aggrecan in degenerative human nucleus pulposus cells: A new therapeutic approach for intervertebral disc disorders. PLoS ONE.

[116] Levicoff E.A., Kim J.S., Sobajima S., Wallach C.J., Larson J.W., Robbins P.D., Xiao X., Juan L., Vadala G., Gilbertson L.G. (2008). Safety assessment of intradiscal gene therapy II: Effect of dosing and vector choice. Spine.

[117] Koepsell L., Zhang L., Neufeld D., Fong H., Deng Y. (2011). Electrospun nanofibrous polycaprolactone scaffolds for tissue engineering of annulus fibrosus. Macromol. Biosci..

[118] Luten J., van Nostrum C.F., De Smedt S.C., Hennink W.E. (2008). Biodegradable polymers as non-viral carriers for plasmid DNA delivery. J. Control. Release.

[119] Srivastava A., Cunningham C., Pandit A., Wall J.G. (2015). Improved gene transfection efficacy and cytocompatibility of multifunctional polyamidoamine-cross-linked hyaluronan particles. Macromol. Biosci..

[120] Feng G., Chen H., Li J., Huang Q., Gupte M.J., Liu H., Song Y., Ge Z. (2015). Gene therapy for nucleus pulposus regeneration by heme oxygenase-1 plasmid DNA carried by mixed polyplex micelles with thermo-responsive heterogeneous coronas. Biomaterials.

[121] Song Q., Jiang K., Zheng D., Jin L., Chen X., Feng Y., Wang K., Han Y., Chen H., Song J. (2023). Programmable DNA hydrogel provides suitable microenvironment for enhancing autophagy-based therapies in intervertebral disc degeneration treatment. J. Nanobiotechnol..

[122] Geers B., Dewitte H., De Smedt S.C., Lentacker I. (2012). Crucial factors and emerging concepts in ultrasound-triggered drug delivery. J. Control. Release.

[123] Wang T., Zhao H., Jing S., Fan Y., Sheng G., Ding Q., Liu C., Wu H., Liu Y. (2023). Magnetofection of miR-21 promoted by electromagnetic field and iron oxide nanoparticles via the p38 MAPK pathway contributes to osteogenesis and angiogenesis for intervertebral fusion. J. Nanobiotechnol..

[124] Gan Y., Li S., Li P., Xu Y., Wang L., Zhao C., Ouyang B., Tu B., Zhang C., Luo L. (2016). A Controlled Release Codelivery System of MSCs Encapsulated in Dextran/Gelatin Hydrogel with TGF-β3-Loaded Nanoparticles for Nucleus Pulposus Regeneration. Stem Cells Int..

[125] Ma T., Liu C., Zhao Q., Zhang Y., Xiao L. (2024). Decellularized nucleus pulposus matrix/chitosan hybrid hydrogel combined with nucleus pulposus stem cells and GDF5-loaded microspheres for intervertebral disc degeneration prevention. Mol. Med..

[126] Feng G., Zhang Z., Dang M., Zhang X., Doleyres Y., Song Y., Chen D., Ma P.X. (2017). Injectable nanofibrous spongy microspheres for NR4A1 plasmid DNA transfection to reverse fibrotic degeneration and support disc regeneration. Biomaterials.

[127] Chang H., Cai F., Zhang Y., Jiang M., Yang X., Qi J., Wang L., Deng L., Cui W., Liu X. (2022). Silencing Gene-Engineered Injectable Hydrogel Microsphere for Regulation of Extracellular Matrix Metabolism Balance. Small Methods.

[128] Wang Y., Kang J., Guo X., Zhu D., Liu M., Yang L., Zhang G., Kang X. (2022). Intervertebral Disc Degeneration Models for Pathophysiology and Regenerative Therapy -Benefits and Limitations. J. Investig. Surg. Off. J. Acad. Surg. Res..

[129] Roberts S., Evans H., Trivedi J., Menage J. (2006). Histology and pathology of the human intervertebral disc. J. Bone Jt. Surg..

[130] Constant C., Hom W.W., Nehrbass D., Carmel E.N., Albers C.E., Deml M.C., Gehweiler D., Lee Y., Hecht A., Grad S. (2022). Comparison and optimization of sheep in vivo intervertebral disc injury model. JOR Spine.

[131] Zhang J., Yu X., Herzog R.W., Samulski R.J., Xiao W. (2021). Flies in the ointment: AAV vector preparations and tumor risk. Mol. Ther. J. Am. Soc. Gene Ther..

[132] Kamatani T., Hagizawa H., Yarimitsu S., Morioka M., Koyamatsu S., Sugimoto M., Kodama J., Yamane J., Ishiguro H., Shichino S. (2022). Human iPS cell-derived cartilaginous tissue spatially and functionally replaces nucleus pulposus. Biomaterials.

[133] Yang K., Song Z., Jia D., Ma J., Huo Y., Zhao Y., Zhang W., Ding W., Wu Z., Yang S. (2022). Comparisons between needle puncture and chondroitinase ABC to induce intervertebral disc degeneration in rabbits. Eur. Spine J..

[134] Tian T., Wang H., Li Z., Yang S., Ding W. (2021). Intervertebral Disc Degeneration Induced by Needle Puncture and Ovariectomy: A Rat Coccygeal Model. BioMed Res. Int..

[135] Vadalà G., Russo F., Pattappa G., Schiuma D., Peroglio M., Benneker L.M., Grad S., Alini M., Denaro V. (2013). The transpedicular approach as an alternative route for intervertebral disc regeneration. Spine.

[136] Vadalà G., De Strobel F., Bernardini M., Denaro L., D’Avella D., Denaro V. (2013). The transpedicular approach for the study of intervertebral disc regeneration strategies: In vivo characterization. Eur. Spine J..

[137] Viswanathan S., Shi Y., Galipeau J., Krampera M., Leblanc K., Martin I., Nolta J., Phinney D.G., Sensebe L. (2019). Mesenchymal stem versus stromal cells: International Society for Cell & Gene Therapy (ISCT^®^) Mesenchymal Stromal Cell committee position statement on nomenclature. Cytotherapy.

[138] Dominici M., Le Blanc K., Mueller I., Slaper-Cortenbach I., Marini F., Krause D., Deans R., Keating A., Prockop D., Horwitz E. (2006). Minimal criteria for defining multipotent mesenchymal stromal cells. The International Society for Cellular Therapy position statement. Cytotherapy.

[139] Hu B.W., Lv X., Chen S.F., Shao Z.W. (2019). Application of Finite Element Analysis for Investigation of Intervertebral Disc Degeneration: From Laboratory to Clinic. Curr. Med. Sci..

